# Weaning Mice and Adult Mice Exhibit Differential Carbon Tetrachloride-Induced Acute Hepatotoxicity

**DOI:** 10.3390/antiox9030201

**Published:** 2020-03-01

**Authors:** Tae Bin Jeong, Doyoung Kwon, Seung Won Son, Sou Hyun Kim, Yun-Hee Lee, Min-Soo Seo, Kil Soo Kim, Young-Suk Jung

**Affiliations:** 1 College of Pharmacy, Pusan National University, Busan 46241, Korea; 2 Department of Cellular and Molecular Pharmacology, University of California San Francisco, San Francisco, CA 94158, USA; 3 College of Pharmacy and Research Institute of Pharmaceutical Sciences, Seoul National University, Seoul 08826, Korea; 4 Laboratory Animal Center, Daegu-Gyeongbuk Medical Innovation Foundation, Daegu 41061, Korea; 5 College of Veterinary Medicine, Kyungpook National University, Daegu 41566, Korea

**Keywords:** drug-induced liver injury, carbon tetrachloride, hepatotoxicity, cytochrome P450, glutathione

## Abstract

Age is a risk factor for drug-induced liver injury (DILI). However, there is a limited understanding of pediatric DILI. Here, 2-week-old weaning and 8-week-old adult male ICR mice were intraperitoneally injected with CCl_4_ (0.1 mmol/kg equal to 15.4 mg/kg) to comparatively evaluate the time-dependent liver damage and cellular events. CCl_4_ significantly enhanced the serum alanine aminotransferase/aspartate aminotransferase levels and hepatic centrilobular necrosis in the weaning mice, whereas it induced mild liver injury in the adult mice. CCl_4_-treated weaning mice exhibited higher hepatic levels of pro-apoptotic proteins (Bax, cleaved caspase-3, -7, and -9), activated MAPKs (p-JNK and p-Erk), and endoplasmic reticulum stress indicators (ATF6 and CHOP) and lower hepatic anti-apoptotic Bcl-2 levels than the adult mice. The weaning mice exhibited enhanced basal hepatic glutathione (GSH) levels due to high glutamate cysteine ligase (GCL) and low anti-cysteine dioxygenase (CDO) enzyme levels. However, CCl_4_ markedly reduced the hepatic GSH levels only in the weaning mice. Furthermore, higher hepatic levels of oxidative stress-induced malondialdehyde, 4-hydroxynonenal, nitrotyrosine-protein adducts, and oxidized proteins were observed in CCl_4_-treated weaning mice than in CCl_4_-treated adult mice. The enhanced levels of hepatic cytochrome P450 (CYP) 2E1 and CYP3A, and decreased hepatic GSH S-transferase (GST)-π and GSH reductase (GR) levels in the weaning mice may contribute to their enhanced susceptibility to liver damage.

## 1. Introduction

The liver is the primary organ for metabolism of xenobiotics, including toxins, environmental pollutants, chemicals, and drugs [1,2]. Xenobiotics can be detoxified and converted to hydrophilic forms for excretion by phase I and phase II drug-metabolizing enzymes in the liver. However, metabolic reactions can also generate reactive products to cause liver damage [2]. 

Drug-induced liver injury (DILI) is the leading cause of acute liver failure and drug withdrawal from the market [3,4]. The risk factors for DILI are age, sex, and genetic polymorphisms [5]. Among the risk factors, age-related changes in hepatic drug metabolism and antioxidant defense system are reported to be associated with the incidence of DILI in different age groups [6,7,8,9].

Although the incidence of DILI among children is reported to be less than 10%, the mortality rate is in the range of 4–31% [4]. The hepatic responses of children to drugs can be markedly different from those of adults mainly due to developmental immaturity [10]. Children are reported to be less sensitive to DILI than the sensitivity exhibited by adults [4]. However, controversial evidence suggests that children are at a greater risk for valproic acid- and propylthiouracil-induced liver injuries than the risk observed in adults subjected to a similar injury [11,12]. There are limited studies on pediatric DILI due to the difficulties in data collection and an insufficient number of research participants [4]. In animal studies, the effects of aging on DILI are examined mostly by comparing the drug-induced hepatotoxic phenotypes of young rodents and old rodents [8,13,14]. However, the susceptibilities of infant animals and juvenile animals to hepatotoxic chemicals/drugs are not fully understood. 

**Figure 1:**
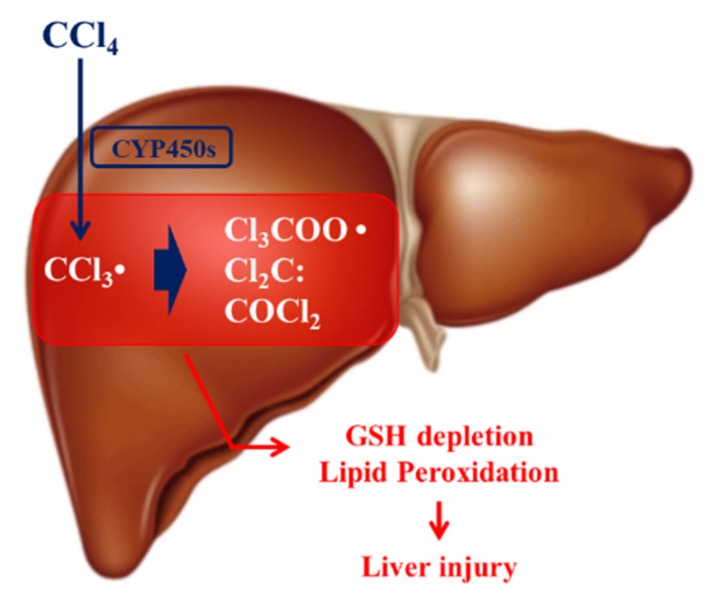
Mechanism of carbon tetrachloride (CCl_4_) hepatotoxicity. CCl_4_ is metabolized by cytochrome P450s (CYP450s) to CCl_3_•, which is further converted into Cl_3_COO•, Cl_2_C, and COCl_2_. These toxic metabolites are detoxified by glutathione (GSH). However, excessive exposure to CCl_4_ causes GSH depletion and oxidative stress via reactions, such as membrane lipid peroxidation that can cause liver damage.

CCl_4_, a well-known experimental hepatotoxin, is reported to cause necrosis, apoptosis, fibrosis, cirrhosis, and cancer in the liver [15,16]. CCl_4_ is mainly metabolized by hepatic cytochrome P450s and is converted into toxic metabolites, such as trichloromethyl radical (CCl_3_•), trichloromethylperoxy radical (CCl_3_OO•), and phosgene (Figure 1), which results in lipid peroxidation that causes hepatocellular damage [15,16]. The reactive metabolites are detoxified non-enzymatically by the cellular antioxidant, glutathione (GSH) or by GSH S-transferase (GST)-catalyzed conjugation reaction [15,16]. However, the excessive generation of free radicals depletes the GSH levels and causes oxidative stress [15,16].

Some rodent studies have investigated the age-related CCl_4_ hepatotoxicity. However, the findings of various rodent studies are contradictory, which may be due to the species- and strain-dependent differences in the hepatic responses [17,18,19,20,21]. Moreover, there are limited studies evaluating the sensitivity of infant/juvenile animals to CCl_4_ hepatotoxicity. In this study, weaning mice and adult mice were acutely intoxicated by a single dose of CCl_4_ to comparatively evaluate the time-dependent severity of liver injury. The weaning mice exhibited more severe CCl_4_-induced liver damages than the one exhibited by the adult mice, that was mainly due to the high levels of hepatic cytochrome P450.

## 2. Materials and Methods 

### 2.1. Animal and Treatments

Male Korl: ICR (referred as ICR) mice were purchased from Koatech (Gyeonggi-do, Korea). The animals were maintained in compliance with the guidelines established and approved by the institutional animal care and use committee at the Pusan National University (PNU-2018-1860). The mice were allowed to acclimatize to the animal facility conditions (room temperature, 22 °C ± 2 °C; humidity, 55% ± 5%; 12 h light-dark cycle) for one week prior to the experiment. Thirty weaning (2-week-old) and thirty young adult (8-week-old) mice were divided into ten groups (six mice per group). After 12 h fasting, the mice were intraperitoneally injected with a single dose of CCl_4_ (0.1 mmol/kg equal to 15.4 mg/kg). The mice were sacrificed at 0 h, 3 h, 6 h, 12 h, and 24 h post-CCl_4_ treatment (six mice per time point). The serum and liver samples were collected for measuring the biochemical parameters. The liver samples stored at −80 °C were used for determining lipid peroxidation/protein oxidation, immunoblotting analysis, and quantifying levels of cysteine, GSH, and taurine. Additionally, some liver samples were fixed in neutral-buffered formalin immediately after the surgery for histopathological examination.

### 2.2. CCl_4_-Induced Hepatotoxicity

The serum sample was collected using the BD Microtainer Serum Collection Tube (BD Life Sciences, Franklin Lakes, NJ, USA). The serum alanine aminotransferase (ALT) and aspartate aminotransferase (AST) levels were measured using the Multiskan GO reader (Thermo Scientific, Waltham, MA, USA), following the method of Reitman and Frankel [22]. Serum total protein and albumin levels were examined with an Automated Chemistry Analyzer (Prestige 24I; Tokyo Boeki Medical System, Tokyo, Japan). The left lateral lobe of liver fixed in neutral-buffered formalin were embedded in paraffin and sectioned. The sections were mounted on the glass slides and stained with hematoxylin and eosin (H&E). The histopathological examination was performed under the Olympus CX41RF microscope (Olympus Co., Tokyo, Japan).

### 2.3. Immunoblotting Analysis

The right posterior lobe of liver was homogenized with chilled ProEX™ CETi Lysis Buffer (TransLab Biosciences, Daejeon, Korea). The protein concentration in the tissue lysates was determined by the bicinchoninic acid (BCA) assay (BioVision Research Products, Mountain View, CA, USA). Equal amounts of proteins were subjected to sodium dodecyl sulfate-polyacrylamide gel electrophoresis (SDS-PAGE). The resolved proteins were transferred onto a nitrocellulose membrane (Bio-Rad Laboratories). The membrane was blocked with 5% skim milk in Tris-buffered saline (pH 7.5) containing 0.2% Tween-20 (TBST) for 1 h at room temperature. Next, the membrane was incubated with the following antibodies for 12 h: anti-cysteine dioxygenase (CDO), anti-glutamate cysteine ligase catalytic subunit (GCLC), anti-GSH peroxidase (GPx), anti-GSH reductase (GR), anti-nitrotyrosine (Santa Cruz Biotechnology, Santa Cruz, CA, USA), anti-CYP2E1, anti-CYP3A (Detroit R&D, Detroit, MI, USA), anti-c-Jun N terminal kinase (JNK), anti-phospho-JNK, anti-extracellular regulated protein kinase (Erk), anti-phospho-Erk, anti-p38, anti-phospho-p38, anti-caspase-3, anti-cleaved caspase-3, anti-caspase-7, anti-cleaved caspase-7, anti-caspase-9, anti-cleaved caspase-9, anti-caspase-12, anti-cleaved caspase-12, anti-poly (ADP-ribose) polymerase (PARP) (Cell Signaling Technology, Danvers, MA, USA), anti-GSH S-transferase-α (GST-α), anti-GST-µ, and anti-GST-π (Detroit R&D). The membrane was washed with TBST and incubated with the appropriate horseradish peroxidase (HRP)-conjugated secondary antibodies. The protein bands were visualized using an enhanced chemiluminescent HRP substrate kit (Western Bright, Advansta, Menlo Park, CA, USA).

### 2.4. Hepatic Lipid Peroxidation Induced by CCl_4_

Hepatic lipid peroxidation was determined by measuring malondialdehyde (MDA) in the thiobarbituric acid reactive substances (TBARS) assay as previously described [23]. The liver homogenate was incubated with 0.2% thiobarbituric acid in 2 M sodium acetate buffer containing 5% butylated hydroxytoluene at 95 °C for 45 min. The reaction mixture was centrifuged. The supernatant was injected into the high-performance liquid chromatography (HPLC) system equipped with a 5-µm Symmetry C18 reversed-phase column (4.6 mm × 150 mm; Eka Chemicals, Bohus, Sweden). The levels of TBARS were monitored by a fluorescence detector (excitation at 515 nm and emission at 553 nm, FLD-3100; Thermo Scientific).

### 2.5. Measurement of Hepatic Protein Oxidation

Protein oxidation assay was performed using OxyBlot ™ (EMD Millipore, Billerica, MA, USA), following the manufacturer’s instructions. The hepatic carbonyl-containing proteins were cross-reacted with 2,4-dinitrophenylhydrazone (DNPH) to produce DNP adducts. After derivatization, equal amounts of proteins were subjected to SDS-PAGE. The oxidized proteins were analyzed by immunoblotting as described in Section 2.3. 

### 2.6. Determination of Levels of Hepatic cysteine, GSH, and Taurine

Hepatic taurine was derivatized with ο-phthalaldehyde/2-mercaptoethanol and quantified using an HPLC system equipped with a fluorescence detector (excitation 338 nm and emission 425 nm; FLD-3100, Thermo Scientific) [24] after separation using a Hector T-C18 column (3 µm × 4.6 mm × 100 mm; Chiral Technology Korea, Daejeon, Korea). The levels of cysteine and GSH were analyzed by the SBD-F derivatization method [25] using an HPLC equipped with a Hector M-C18 column (3 µm × 4.6 mm × 150 mm: Chiral Technology Korea) and a fluorescence detector (excitation 385 nm and emission 515 nm; FLD-3100, Thermo Scientific).

### 2.7. Statistical Analysis

All data have been expressed as mean ± standard deviation (SD). The data were analyzed by two-tailed unpaired Student’s *t*-test. The difference was considered to be statistically significant if the *p*-value was found to be less than 0.05.

## 3. Results

### 3.1. CCl_4_-Induced Liver Injuries in Weaning Mice and Adult Mice

**Figure 2:**
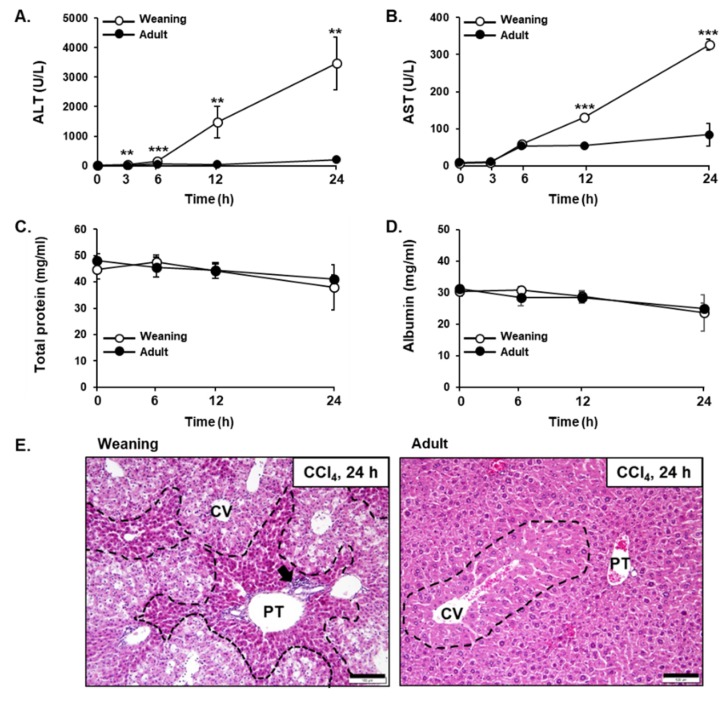
Carbon tetrachloride (CCl_4_)-induced liver injury in the weaning mice and adult mice. The serum levels of (**A**) alanine aminotransferase (ALT), (**B**) aspartate aminotransferase (AST), (**C**) total protein, and (**D**) albumin. Male mice were injected with CCl_4_ (0.1 mmol/kg equal to 15.4 mg/kg, intraperitoneal (IP)) and each parameter was measured at indicated time point after CCl_4_ treatment. The results have been presented as mean ± standard deviation (SD). **, *** Significantly different from the corresponding weaning mice (Student’s *t*-test, *p* < 0.01 and *p* < 0.001, respectively). (**E**) The histopathological analysis of hematoxylin and eosin (H&E)-stained liver tissues at 24 h post-CCl_4_ treatment. Black dashed line, degeneration area; Black arrow, inflammatory cells; CV, central vein; PT, portal triad. Scale bar, 100 μm.

The weaning mice and adult mice exhibited differential susceptibility to CCl_4_ hepatotoxicity. The serum levels of ALT and AST, which indicate liver injury, in the weaning mice were found to be significantly higher than those in the adult mice post-CCl_4_ administration (Figure 2A,B). Total protein and albumin levels in serum, reflecting the biosynthetic function of the liver, did not show a significant difference in age-dependent changes as well as time-dependent changes for 24 h after CCl_4_ treatment (Figure 2C,D). The analysis of H&E-stained liver tissues indicated that the weaning mouse liver exhibited severe centrilobular necrosis at 24 h post-CCl_4_ injection, whereas the adult mouse liver exhibited mild liver injury (Figure 2E). This suggested that the weaning mice were more susceptible to CCl_4_-induced hepatotoxicity than the susceptibility observed in adult mice. 

### 3.2. CCl_4_-Induced Hepatic Apoptosis and Endoplasmic Reticulum (ER) Stress in Weaning Mice and Adult Mice

**Figure 3:**
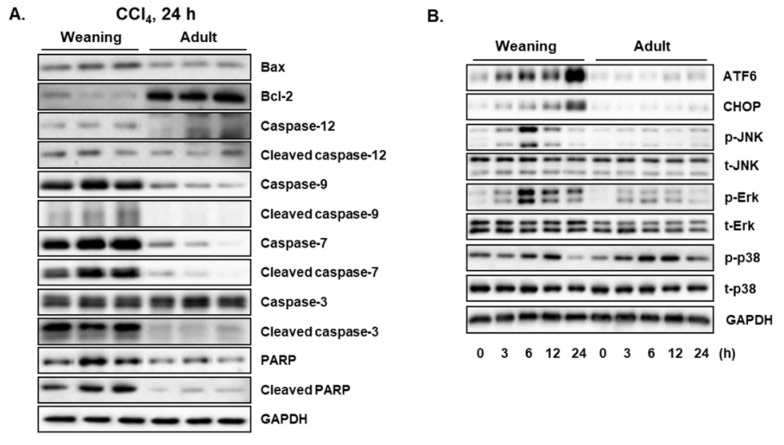
Carbon tetrachloride (CCl_4_)-induced hepatic apoptosis and endoplasmic reticulum (ER) stress in the weaning mice and adult mice. (**A**) Proteins involved in the apoptotic signaling pathway were measured at 24 h post-CCl_4_ injection. (**B**) Time-dependent changes in the expression levels of ER stress markers and MAPKs. Male mice were injected with CCl_4_ (0.1 mmol/kg equal to 15.4 mg/kg, intraperitoneal (IP)). The liver tissues were obtained at 0 h, 3 h, 6 h, 12 h, and 24 h post-CCL_4_ injection. Hepatic proteins were detected by immunoblotting and GAPDH was used as the loading control.

CCl_4_ is reported to induce hepatic cell death by apoptosis [15,26]. To comparatively evaluate the CCl_4_-induced hepatic apoptosis between weaning mice and adult mice, the expression levels of proteins involved in the apoptotic signaling pathway were determined at 24 h post-CCl_4_ injection. The weaning mice exhibited higher pro-apoptotic Bax hepatic levels and lower anti-apoptotic Bcl-2 hepatic levels than the ones exhibited by the adult mice (Figure 3A). Compared to the adult mice, the weaning mice exhibited higher hepatic levels of caspase-9 (initiator caspase) and caspase-7 (executioner caspase) and their active cleaved forms (Figure 3A). Although the hepatic levels of caspase-3 were not markedly different between the two groups, the weaning mice exhibited higher hepatic levels of cleaved caspase 3 (active form) than the levels exhibited by the adult mice (Figure 3A). The hepatic levels of PARP, which is cleaved by caspases, in the weaning mice were found to be higher than those in the adult mice (Figure 3A). These results indicate that CCl_4_ induces higher hepatic apoptosis in the weaning mice than the one induced in the adult mice. Endoplasmic reticulum (ER) stress is a cellular condition of disturbed ER homeostasis, which is characterized by the accumulation of unfolded proteins and misfolded proteins in the ER [27,28]. During ER stress, unfolded protein responses (UPRs) are activated to restore the ER functions. However, prolonged ER stress promotes apoptosis to remove the damaged cells [27,28]. Activating transcription factor 6 (ATF6) is a UPR sensor protein that is induced and activated in response to ER stress [27,28]. Treatment with CCl_4_ in a time-dependent manner increased the hepatic levels of C/EBP homologous protein (CHOP), an indicator of ER stress, and ATF6 in the weaning mice after CCl_4_ treatment. However, CCl_4_ treatment did not markedly affect the hepatic levels of CHOP and ATF6 in the adult mice (Figure 3B). Cellular stresses activate mitogen-activated protein kinases (MAPKs), which mediate the intracellular signaling involved in cell proliferation, differentiation, survival, and apoptosis [29,30]. Three MAPKs, namely JNK, Erk, and p38, are reported to be activated in response to toxic stimuli, such as oxidative stress and inflammation [29,30]. Treatment with CCl_4_ increased the hepatic levels of activated/phosphorylated forms of JNK, Erk, and p38 in both weaning mice and adult mice (Figure 3B). However, the weaning mice exhibited higher hepatic p-JNK and p-Erk levels than the ones exhibited by the adult mice. There was no significant difference in the hepatic p-p38 levels between the two groups. ER stress can activate JNK, which subsequently triggers apoptosis [27,28]. Thus, the enhanced activation of JNK in weaning mice may explain the ER stress-induced apoptotic cell death in the livers.

### 3.3. CCl_4_-Induced Oxidative Stress in the Liver of Weaning Mice and Adult Mice

**Figure 4:**
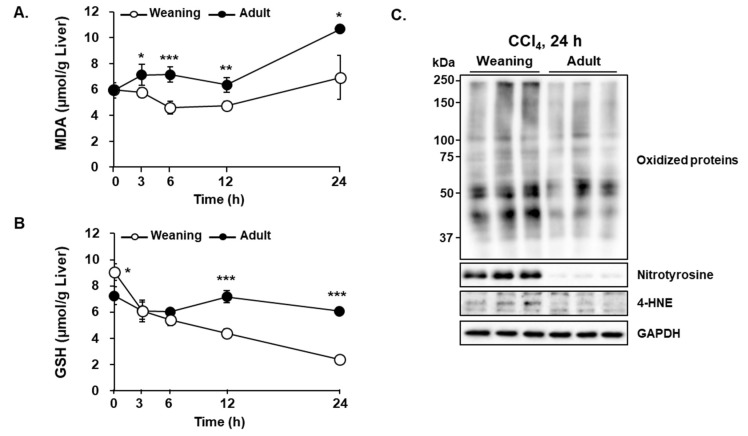
Carbon tetrachloride (CCl_4_)-induced oxidative stress in the liver of weaning mice and adult mice. Time-dependent changes in the (**A**) hepatic malondialdehyde (MDA) and (**B**) hepatic glutathione (GSH) levels. (**C**) The levels of oxidized proteins, nitrotyrosine-protein adducts, 4-hydroxynonenal (4-HNE) at 24 h post-CCl_4_ treatment. Male mice were injected with CCl_4_ (0.1 mmol/kg equal to 15.4 mg/kg, intraperitoneal (IP)). The liver tissues were obtained at 0 h, 3 h, 6 h, 12 h, and 24 h post-CCl_4_ treatment. The results have been presented as mean ± standard deviation (SD). *, **, *** Significantly different from the corresponding weaning mice (Student’s *t*-test, *p* < 0.05, *p* < 0.01, and *p* < 0.001, respectively).

Oxidative damages of cellular molecules are reported to be the main mechanism underlying CCl_4_-mediated hepatotoxicity [15,16]. The reactive radicals generated from CCl_4_ not only oxidize proteins, lipids, and DNA but also cause GSH depletion, which exacerbates oxidative stress [15,16]. Treatment with CCl_4_ enhanced the hepatic levels of MDA, a lipid peroxidation end product of polyunsaturated fatty acids (PUFAs), in the weaning mice but not in the adult mice (Figure 4A). The hepatic GSH level in the weaning mice was higher than that in the adult mice prior to CCl_4_ injection (Figure 4B). However, CCl_4_ decreased the hepatic GSH levels in the weaning mice in a time-dependent manner. The hepatic GSH levels in the adult mice were slightly decreased upon CCl_4_ administration (Figure 4B). The hepatic levels of oxidized proteins, 4-hydroxynonenal (HNE), and nitrotyrosine-protein adducts in the weaning mice were higher than those in the adult mice (Figure 4C). HNE, another lipid peroxidation product from PUFAs, and protein tyrosine residues are nitrated by peroxynitrite (ONOO^−^), which is produced by the reaction between superoxide anion and nitric oxide. Thus, the severity of oxidative stress induced by CCl_4_ appears to be closely associated with the differential susceptibility to hepatotoxicity between the two groups.

### 3.4. Hepatic CYP Levels in Weaning Mice and Adult Mice

**Figure 5:**
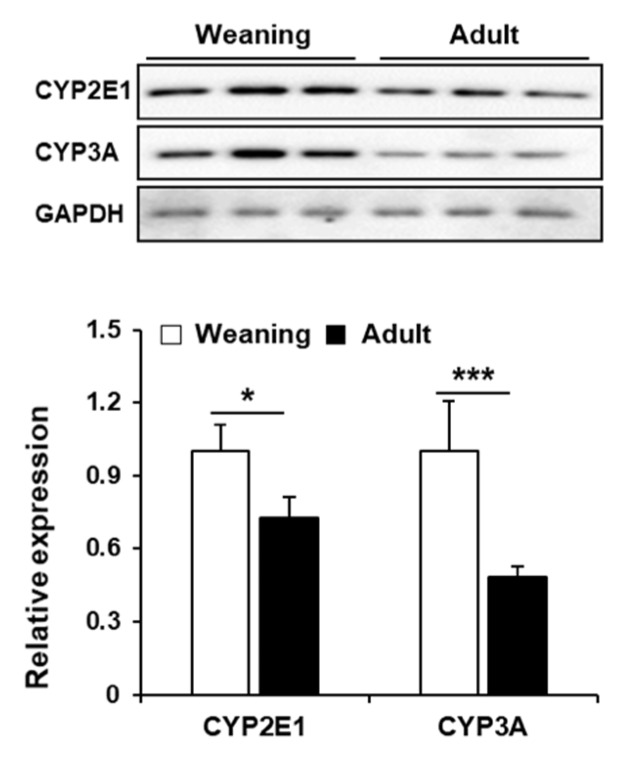
Hepatic CYP2E1 and CYP3A levels in the weaning mice and adult mice without carbon tetrachloride (CCl_4_) treatment. Hepatic CYPs were detected by immunoblotting and GAPDH was used as the loading control. The relative protein amounts are presented as mean ± standard deviation (SD). *, *** Significantly different from the corresponding weaning mice (Student’s *t*-test, *p* < 0.05 and *p* < 0.001, respectively).

The major CYP isozymes for CCl_4_ metabolism are CYP2E1 and CYP3A. The weaning mice exhibited higher hepatic CYP2E1 and CYP3A levels than the levels exhibited by the adult mice (Figure 5). This suggested that the enhanced susceptibility of weaning mice to CCl_4_ may be due to the enhanced metabolic activation of CCl_4_ via the CYPs.

### 3.5. Hepatic Enzymes Involved in GSH Metabolism in Weaning Mice and Adult Mice

**Figure 6:**
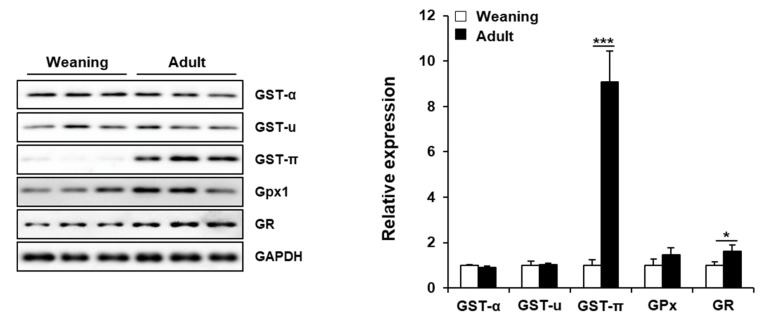
Hepatic proteins involved in glutathione (GSH) metabolism in the weaning mice and adult mice without carbon tetrachloride (CCl_4_) treatment. Hepatic proteins were detected by immunoblotting and GAPDH was used as the loading control. The relative protein amounts have been presented as mean ± standard deviation (SD). *, *** Significantly different from the corresponding weaning mice (Student’s *t*-test, *p* < 0.05 and *p* < 0.001, respectively).

GSTs catalyze the detoxification reaction by conjugating GSH with the electrophiles [31]. The hepatic levels of GST-α and GST-µ classes were similar between the weaning mice and adult mice. However, the hepatic levels of GST-π class in the adult mice were about 9-fold higher than those in the weaning mice. This suggested that the liver of adult mice may exhibit higher GST-π-mediated detoxification, that can also be a reason for the lower basal level of GSH in these mice as GSH is consumed by GSTs. The levels of GPx, which reduces lipid peroxides and H_2_O_2_ using GSH [32], were significantly different between the two groups. However, the hepatic levels of GR, which converts oxidized GSH (GSSG) to reduced GSH [32], in the weaning mice were lower than those in the adult mice. This can also explain the enhanced vulnerability of weaning mice to oxidative stress (Figure 6).

### 3.6. Hepatic Synthesis of GSH and Taurine from Cysteine in Weaning Mice and Adult Mice

**Figure 7:**
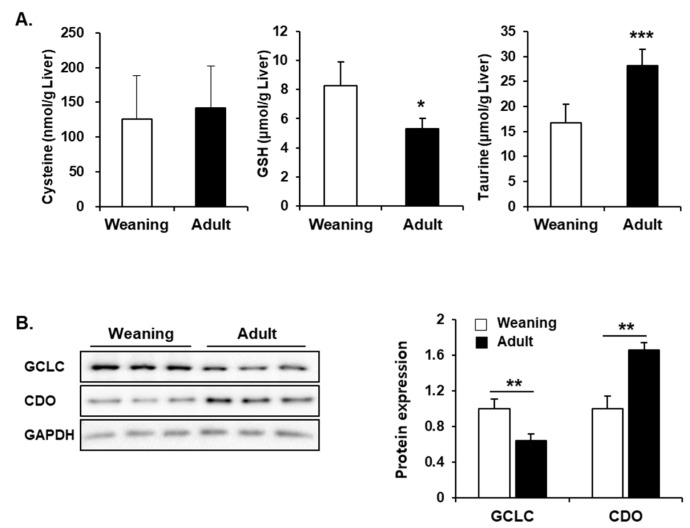
Hepatic levels of glutathione (GSH), taurine, and the respective synthetic enzymes in the weaning mice and adult mice without carbon tetrachloride (CCl_4_) treatment. (**A**) Hepatic levels of cysteine, GSH, and taurine. (**B**) Relative glutamate cysteine ligase catalytic subunit (GCLC) and cysteine dioxygenase (CDO) protein levels. The results have been presented as mean ± standard deviation (SD). *, **, *** Significantly different from the corresponding weaning mice (Student’s *t*-test, *p* < 0.05, *p* < 0.01, and *p* < 0.001, respectively).

As described previously, the weaning mice exhibited higher hepatic GSH levels that the adult mice even without CCl_4_ treatment (Figure 4B and Figure 7A). To examine the GSH synthetic pathway, the hepatic levels of cysteine (precursor of GSH) and GCLC (rate-limiting enzyme for GSH synthesis) were comparatively evaluated between the weaning mice and adult mice. There was no significant difference in the hepatic cysteine level between the two groups. The hepatic GCLC levels in the weaning mice were higher than those in the adult mice (Figure 7B). Interestingly, the hepatic levels of taurine (another metabolic product from cysteine) and CDO (the first enzyme utilizing cysteine for taurine synthesis) in the weaning mice were significantly lower than those in the adult mice (Figure 7A,B). Therefore, the higher rate of GSH synthesis and the lower consumption of cysteine for taurine production may contribute to the enhanced hepatic GSH level in the weaning mice.

## 4. Discussion

Aging is reported to be a risk factor for DILI. Compared to the adults, children are less sensitive to DILI because of the lower CYP-mediated metabolic rates and higher GSH synthetic rate in the liver [4,33]. However, the database collected from 1997 to 2012 by FDA’s Adverse Event Reporting System show that the clinical incidences of liver injury in children (0 year–9 years, 187 cases; 10 years–19 years, 236 cases) induced by methotrexate is higher than those in young adults (20 years–29 years, 120 cases) [34]. Moreover, lower blood GSH level in <1-year-old individuals than that in older age (2 to 40 years) groups was found in a research of Turkish hospital [35]. These suggest that infants and children are also at a risk for DILI. CCl_4_-induced hepatotoxicity in <20-day-old mice is more severe than that in the adult mice [17,19]. The underlying reason for the differential hepatic sensitivity has been not elucidated. In this study, we comparatively evaluated CCl_4_-induced hepatotoxicity between 14-day-old mice, that represents infant age before weaning, and 8-week-old mice that represents adulthood. The results indicated that the differential hepatic levels of CYPs, GST-π, and GR proteins can potentially contribute to the differential susceptibility to CCl_4_ between the weaning mice and adult mice. 

CYPs, which are heme-containing monooxygenases, are the major hepatic phase I drug-metabolizing enzymes. CYPs have an important role in xenobiotic biotransformation and excretion. However, CYPs can also have a deleterious role in the metabolic activation of diverse chemicals/drugs, such as carbon tetrachloride, alcohol, acetaminophen, and halothane [2,4]. Among the 57 isozymes of human CYPs, CYP2E1 and CYP3A are mainly responsible for mediating the hepatotoxicity of CCl_4_ [15,36,37]. CYP-mediated reductive cleavage of a carbon-chlorine bond of CCl_4_ results in the generation of noxious trichloromethyl radical (CCl_3_•), which is further converted into trichloromethylperoxy radical (CCl_3_OO•) upon reaction with oxygen [15]. During the catalytic cycle of CYPs, reactive oxygen species (ROS), such as superoxide anion (O_2_^−^•) and hydrogen peroxide (H_2_O_2_) are generated that can also induce oxidative stress [38]. In this study, CCl_4_-treated weaning mice exhibited enhanced hepatic necrosis (Figure 3) and apoptosis (Figure 3), which was due to the induction of oxidative stress in the mice livers as evidenced by enhanced lipid peroxidation and protein oxidation, as well as decreased GSH levels (Figure 4). However, CCl_4_-treated adult mice exhibited mild liver injury. Higher susceptibility of weaning mice to CCl_4_ may be attributed mainly to higher protein levels of CYP2E1 and CYP3A (Figure 5). The extensive centrilobular necrosis observed in the CCl_4_-injected weaning mouse livers also supports the role of CYPs in mediating CCl_4_-induced hepatotoxicity. The centrilobular region exhibits higher expression levels of CYPs than the levels exhibited by the periportal region [39,40]. 

Several clinical and animal studies have demonstrated the age-related changes in the hepatic levels of CYPs. An animal study demonstrated that hepatic activities of CYP2B and CYP3A in 6 week-old rats were significantly higher than those in the 21-month-old rats [41]. Declines of hepatic CYP2E1 and/or CYP3A correlated with the alleviations of acetaminophen- and isoniazid-induced hepatotoxicities with aging have been reported in rodents [13,14,42] as similar as our results (Figure 5). In contrast to our results, human liver CYP2E1 and CYP3A4 gradually increased after birth, while CYP3A7 decreased [43,44], suggesting that human CYP system in infant functions more slowly than in adult. However, only limited research in humans has been reported and thus it is not clear whether there is species difference in CYP development.

Apoptosis is a programmed and self-destructive cell death characterized by cellular condensation and cellular shrinkage. In contrast, necrosis is characterized by immediate and uncontrolled cell death accompanied by cell lysis. Apoptosis is reported to be an early process of CCl_4_-induced hepatic cell death, which is followed by necrosis [26,45]. In studies using mouse models, acute administration of CCl_4_ increased the levels of activated caspase-3, -9, and -12 with the release of cytochrome c in the liver [26,45]. Caspase-12-deficient mice exhibited markedly mitigated CCl_4_-induced apoptosis and liver injury when compared to the wild type mice. This indicated that apoptosis is also an important mechanism of cell death in CCl_4_-treated liver [26]. These studies demonstrated that CCl_4_-induced ER stress, which is indicated by the upregulated expression of GRP78, CHOP, IRE1, and XBP1, is the mechanism underlying apoptosis [26,45]. As CYPs are mainly localized at the hepatocyte ER, reactive CCl_4_ metabolites and ROS generated in this organelle can perturb ER calcium homeostasis and protein folding function, which results in ER stress [26]. In this study, CCl_4_ treatment increased the levels of CHOP in a time-dependent manner, that is upregulated by ATF6, in the weaning mice (Figure 3). This indicated that CCL_4_ induced ER stress in the liver. CHOP, a pro-apoptotic transcription factor, down-regulates levels of Bcl-2 and JNK. JNK is activated by ER stress, which results in the phosphorylation of Bcl-2 and the release of Bax [27,28]. The free Bax can form pores on the ER and mitochondrial membranes resulting in the release of ER calcium and mitochondrial cytochrome c, which mediates the activation of caspases [27,28]. Thus, the increased levels of ATF6, CHOP, p-JNK, and activated caspase suggest the association of ER stress and apoptosis in the livers of CCl_4_-treated weaning mice. However, the adult mice appear to be relatively resistant to CCl_4_-induced ER stress and apoptosis as evidenced by the unaltered levels of ER stress markers and high levels of anti-apoptotic Bcl-2 level in the liver. 

GSH (γ-L-glutamyl-L-cysteinylglycine) is an essential non-protein thiol that has crucial roles in the detoxification of xenobiotics and the inhibition of oxidative stress by neutralizing free radicals and ROS in the liver [32]. In this study, the weaning mice exhibited high hepatotoxicity in spite of high hepatic GSH level after CCl_4_ administration. This indicated that hepatic CYPs, rather than GSH, can be the primary factor for liver damage. The low levels of GST-π and GR proteins in the weaning mouse livers cannot be ruled out as the potential reasons for greater sensitivity as these enzymes are important for GSH conjugation and GSH regeneration from oxidized GSH (GSSG), respectively. GSTs are phase II detoxification enzymes that catalyze the thioether conjugation reaction of GSH with electrophiles [31]. The hepatic GST levels are reported to gradually increase from 2 weeks to 23 months of age in rats [41,46]. Xu et al. demonstrated that rat hepatic GST-π protein and mRNA expression continuously increased from 14 days up to 2 years after birth [47], which concurred with the results of our study. GST-π is reported to inhibit JNK phosphorylation by interacting with the C-terminus of JNK [48,49]. Moreover, oxidative stress dissociates the JNK-GST-π complex due to the oligomerization of GST-π, which results in the loss of JNK inhibition and subsequently apoptosis [48,50]. The enhanced expression of hepatic GST-π in the adult mice can partly contribute to the decreased activation of JNK. Thus, the adult mice exhibited higher resistance to apoptosis than the resistance exhibited by the weaning mice. 

Age-related decline of GSH levels is reported in mammalian cells, which increases the risk of oxidative tissue damages in the elderly [51]. The main reason for the declined GSH levels is reported to be the reduction of de novo synthesis of GSH in aged cells [51]. GCL, the rate-limiting enzyme for GSH biosynthesis, condenses cysteine and glutamic acid to produce γ-glutamylcysteine, which reacts with glycine in the presence of GSH synthase to form GSH [32]. Older animals are reported to exhibit lower hepatic GCL activity due to the down-regulation of GCL expression [52,53,54]. CDO is another major enzyme consuming cysteine for taurine synthesis [55]. This enzyme adds dioxygen to cysteine thiol to form cysteine sulfinate, which is further converted to taurine [55]. The metabolic fate of cysteine depends on the activities of CDO and GCL. Thus, the cellular GSH concentration can also be affected by CDO in the liver. Previously, the levels of hepatic taurine were reported to decrease with aging due to the decreased CDO activity in middle-aged (9 months-10 months) and old (26 months-28 months) rats. However, the levels of hepatic taurine were not determined in the younger animals before they reached adulthood [56,57]. Our study, for the first time, demonstrates that infant mice have lower hepatic CDO level, that decreases the taurine concentration, than hepatic CDO levels observed in the adult mice (Figure 7). The low levels of CDO and high levels of GCL proteins in the weaning mouse livers imply that cysteine may be more efficiently utilized for the synthesis of GSH rather than the synthesis of taurine, that appears to contribute to the enhanced GSH levels in the weaning mice.

## 5. Conclusions

**Figure 8:**
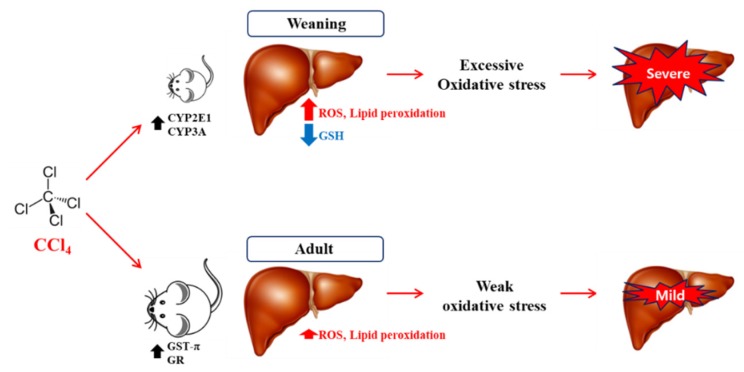
Summary and hypothetical mechanisms of age-related susceptibility to carbon tetrachloride (CCl_4_) hepatotoxicity in weaning mice and adult mice. Higher metabolic rate of CCl_4_ by hepatic CYP2E1 and CYP3A in weaning mice can cause excessive oxidative stress via reactive oxygen species (ROS) generation and glutathione (GSH) depletion, which can lead to significant liver injury. However, adult mice exhibit less CYP levels and high levels of GST-π and GR in their livers. This can contribute to resistance to CCl_4_ hepatotoxicity.

The results of this study have been summarized in Figure 8, that illustrates that weaning mice were found to be more vulnerable to CCl_4_-induced liver injury than the extent of damage observed in the adult mice. The enhanced CYP2E1 and CYP3A protein levels in the weaning mice may contribute to severe necrosis and apoptosis through cellular dysfunctions induced by oxidative and ER stresses in the liver. Interestingly, the high GSH level and GSH synthesis rate in the liver of weaning mice cannot inhibit the CCl_4_-mediated hepatotoxicity, that also indicates that CYPs are the major contributors to liver injury. Therefore, hepatic CYP activities in infants/children should be carefully considered for prescribing medication to prevent DILI. These findings demonstrated the possible reasons for the differential susceptibility of infant animals and adult animals using the CCl_4_ hepatotoxicity model. The limitation of this study is that it does account for the species-dependent differences in the drug metabolism and antioxidant defense systems. Thus, further studies will be needed for a detailed understanding of pediatric DILI.
